# Healthcare personnel at non–acute-care facilities are at risk of COVID-19 from workplace and community exposures

**DOI:** 10.1017/ash.2023.217

**Published:** 2023-09-29

**Authors:** Armaghan-e-Rehman Mansoor, Caroline O’Neil, David McDonald, Victoria Fraser, Hilary Babcock, Jennie Kwon

## Abstract

**Background:** Healthcare personnel (HCP) working in non–acute-care facilities are at high risk of COVID-19. We sought to determine SARS-CoV-2 seroprevalence, and analyzed behaviors and activities related to COVID-19 acquisition in this cohort. **Methods:** Between May and June 2021, HCP were enrolled at a skilled nursing facility and a memory care facility in St. Louis, Missouri. Data regarding demographics, prior SARS-CoV-2 testing, symptoms consistent with COVID-19 in the previous 6 months, COVID-19 vaccination, personal protective equipment (PPE) use, and COVID-19 exposures were collected via survey. Blood specimens were obtained to determine SARS-CoV-2 nucleocapsid IgG antibody seroprevalence (Abbott Laboratories). Study protocol was approved by the Washington University Institutional Review Board. **Results:** The survey was completed by 74 HCP. 82% of participants were female, and 31% reported >10 years of healthcare experience. The overall SARS-CoV-2 seropositivity rate was 8.9% (5 HCP). Of the surveyed HCP, 50% reported symptoms concerning for COVID-19 in the prior 6 months. Headache (38%), fatigue (35%) and fever (27%) were the most common self-reported symptoms. Among symptomatic HCP, only 35% sought medical care for these symptoms. All HCP reported having taken at least 1 COVID-19 test prior to study enrollment. Of note, 18.9% (14 HCP) had a self-reported prior positive SARS-CoV-2 PCR test, of whom 9 HCP were seronegative. All seronegative HCP with a self-reported history of COVID-19 reported infection >3 months before study participation. Completion of a primary COVID-19 vaccination series was reported by 86% of HCP. Known exposure to COVID-19 at work was reported by 28% of HCP. When asked about PPE at the time of workplace exposure, N95 mask use was reported by 81%, gloves by 57%, gowns by 33%, face shields by 29% and surgical masks by 14%. Known specific exposure to COVID-19 outside work was reported by 31% of HCP. **Conclusions:** One year after the initial COVID-19 pandemic impacted the St. Louis region, HCP at non–acute-care facilities had a SARS-CoV-2 seroprevalence of 8.9%. Similar frequency of exposures were reported from both the workplace and community, with high rates of PPE use at the workplace. HCP in such settings remain at high risk of COVID-19 exposure from workplace and community exposures. Ongoing efforts are needed to maintain PPE use to prevent SARS-CoV-2 transmission within non–acute-care facilities, and continue access to timely COVID-19 screening for HCP.

**Disclosure:** None

